# Supplementary material for: Structure and Photoluminescence Properties of EuSiN2
Source: Chemistry. 2025 Apr 29;31(29):e202404395. doi: 10.1002/chem.202404395 (PMC12099192; doi:10.1002/chem.202404395)
Supplement: Supplementary file 1 — Supporting Information [file CHEM-31-e202404395-s001.docx]

**Structure and Photoluminescence Properties of EuSiN_2_**

Cordula Braun,^[a]*^ Senthil Kumar Kuppusamy,^[b]^ Pascal Uhlemann,^[c,d]^

Peter Höhn^[d]^

[a] ^*^ Dr. C. Braun,

Karlsruhe Institute of Technology (KIT)

Institute for Applied Materials (IAM)

Herrmann-von-Helmholtz-Platz 1,

D-76344 Eggenstein-Leopoldshafen

E-mail: [Cordula.Braun@kit.edu](mailto:Cordula.Braun@kit.edu)

[b] Dr. S. Kuppusamy,

Karlsruhe Institute of Technology (KIT)

Institute of Quantum materials and Technologies (IQMT)

Herrmann-von-Helmholtz-Platz 1,

D-76344 Eggenstein-Leopoldshafen

[c] P. Uhlemann,

Forschungszentrum Jülich GmbH

Institute of Fusion Energy and Nuclear Waste Management (IFN)

Nuclear Waste Management (IFN-2)

Wilhelm-Johnen-Straße

D-52428 Jülich (current adress)

[d] P. Uhlemann, Dr. P. Höhn,

Max-Planck-Institute for Chemical Physics of Solids,

Nöthnitzer Straße 40,

D-01187 Dresden

**Abstract**

With the europium nitridosilicate, EuSiN_2_ a novel member of the multifaceted *M*SiN_2_ family is presented and compared with the structural similar compounds *M*SiN_2_ (*M* = Ca, Sr, Ba). A different way of presenting the structure as *M*-N polyhedra layers is introduced.

The crystal structure of EuSiN_2_, being isotypical to SrSiN_2_, was determined and refined based on powder X-ray diffraction data in space group *P*2_1_*/c* (no. 14) with *a* = 5.96381(2)Å, *b* = 7.25982(3)Å, *c* = 5.488338(19)Å, *β* = 113.33°, *V* = 218.18(5)Å^3^ and *Z* = 4.

As the Eu^2+^-doped *M*SiN_2_ compounds show excellent luminescence properties as LED phosphors, the luminescence behavior of dark red emitting EuSiN_2_ has been investigated as well. Two emission peaks are visible at 708 nm and 764 nm.

Many europium doped nitridosilicates show more or less developed shoulders for their luminescence peaks, but two clearly shaped emission peaks are a novel feature for nitridosilicates.

Comprehensive and detailed comparisons are given and discussed based on works in literature.

The chromaticity coordinate positions of EuSiN_2_ in the CIE 1931 diagram are *x* = 0.678 and *y* = 0.284.

**Keywords:** Luminescence • Nitridosilicates • Rare Earth • Europium (II) • NLO Materials

***Introduction***

Rare-earth activated nitridosilicate phosphors are attractive materials for phosphor-converted white light-emitting diodes (pc-LEDs), because of their auspicious chemical and physical properties.^[1,2]^ Their wide-ranging applicability can be ascribed to their significantly extended structural varieties built on covalent SiN_4_ network structures providing possible migration pathways^[3–5]^ for cation exchange and their excellent thermal, chemical, and mechanical stability. An extensive series of *M*SiN_2_ compounds (*M* = Be, Mg, Ca, Sr, Ba, Zn)^[6,7,16–18,8–15]^ is already known in literature, standing out due to their multifaceted application in industry as e.g. sinter additives for Si_3_N_4_ high performance ceramics^[19–21]^  or Eu^2+^-doped LED phosphors, showing excellent luminescence properties with a typical broadband emission for Eu^2+^ in the orange-red region.^[20,22,31–33,23–30]^ The luminescence properties of Eu^2+^-doped *M*SiN_2_ (*M* = Mg, Ca, Sr, Ba) are influenced by the crystal structure of the host lattice, the degree of covalency, the crystal field environment, the size of the cation, the crystallographic *M* site and the coordination of its next neighbours. The increase of the Eu^2+^ concentration causes a red shift of the emission peaks of *M*SiN_2_ (*M* = Sr, Ba). However a fine tuning of the luminescence properties can be influenced by the respective Sr/Ba-ratio (at fixed Eu^2+^ concentration).^[29,30]^

Beyond the luminescent aspect, polymeric non-metallic nitrides are considered as well as very important materials in the development of high-performance devices for optoelectronic applications. Crucial here are bulk electronic properties such as band gaps, high thermal conductivity and low electrical conductivity, and in this context the *M*-Si-N compounds (*M* = Mg, Ca, Sr, Ba, Zn) have been studied comprehensively.^[7,11]^

The combination of nitridosilicate anionic host structures with *M*^2+^ and *M*^3+^ cations was an unknown phenomenon till Huppertz *et al.*^[34]^ reported on the first nitridosilicate (EuYbSi_4_N_7_), comprising Eu^2+^ and Yb^3+^ cations. A few years later Hintzen *et al.*^[35]^ published the isostructural compound EuYSi_4_N_7_. Eu_2_SiN_3,_^[36]^ a mixed valenced (Eu^2+^ and Eu^3+^) nitridosilicate, is a chain-type silicate comprising one-dimensional infinite nonbranched *zweier* chains of corner-sharing SiN_4_-tetrahedra. There are two crystallographically distinct europium sites at two different Wyckoff positions being occupied with Eu^2+^ and Eu^3+^, respectively.

*M*_2_Si_5_N_8_:Eu^2+^ (red-orange, 2-5-8 phosphors) and *M*Si_2_O_2_N_2_:Eu^2+^ (yellow-green,1-2-2-2 phosphors) (*M* = Ca, Sr, Ba) were significant discoveries in the field of Eu doped phosphors.^[33,37,46–50,38–45]^

Also Eu_2_Si_5_N_8_ has been discovered long before and was already subject of multiple investigations, as e.g. magnetic susceptibility measurements, DFT and optoelectronic investigations,^[34,51–53]^ however no detailed luminescence studies can be found in literature except the luminescence spectra of Eu_2_Si_5_N_8_ Huppertz showed in his dissertation.^[53]^

Herein, we present a novel europium nitridosilicate, namely EuSiN_2_, together with its luminescence properties and a detailed structure comparison with the related compounds *M*SiN_2_ (*M* = Ca, Sr, Ba).

***Results***

***Synthesis and Structure characterization***

EuSiN_2_ was synthesized from EuN and β-Si_3_N_4_ (Chempur 99.999%) according to the reaction in eq. (1). Hereby a dark red powder was obtained. For detailed information see experimental part.

3 EuN + Si_3_N_4_ 3 EuSiN_2_ + ^1^/_2_ N_2_ (Eq. (1))

1550 °C

N_2_

The crystal structure of EuSiN_2_ was determined and refined based on X-ray powder diffraction data in space group *P*2_1_*/c* (no. 14) with *a* = 5.96381(2)Å, *b* = 7.25982(3)Å, *c* = 5.488338(19)Å, *β* = 113.33°, *V* = 218.18(5)Å^3^ and *Z* = 4.

The X-ray measurements were performed on a STOE STADI P powder diffractometer (see Table 1 and experimental part). The sample, which was slightly air sensitive, was enclosed and protected against atmosphere in a glass capillary with 0.3 mm diameter. Figure 1 shows the observed and calculated X-ray powder diffraction pattern as well as their difference curve after Rietveld refinement.^[54]^ Crystallographic data and details of the Rietveld refinement are listed in Table 1. The occupied Wyckoff sites and refined atomic coordinates of EuSiN_2_ are displayed in Table 2. For selected bond lengths of EuSiN_2_ see Table 3. Estimated standard deviations are calculated in agreement with *ref.*^[55]^

Table 1: Crystallographic data for EuSiN_2_, (standard deviation in parentheses).

| Formula | EuSiN_2_ |
| --- | --- |
| Formula mass / g mol^-1^ | 208.06 |
| Crystal system | monoclinic |
| Space group | P2_1_*/c* (no. 14) |
| Cell parameters / Å | *a* = 5.96381(2) |
|  | *b =* 7.25982(3) |
|  | *c* = 5.488338(19) |
|  | *β* = 113.33° |
| Cell volume / Å^3^ | 218.18(5) |
| Formula units / cell | 4 |
| Diffractometer | STOE STADI P |
| Radiation / Å | Co-Kα_1_ (λ = 1.788965Å) |
| Monochromator | Ge 111 (curved) |
| Temperature / K | 293 |
| Data range (2θ),  step width | 4° ≤ 2θ ≤ 85°, 0.01° |
| Structure refinement | Rietveld refinement, Fullprof^[54]^ |
| Background treatment | 25 fixed background points |
| Profile function | Pseudo-Voigt (no. 7) |
| *R_Bragg_* | 3.06 |
| *R_value_* | 1.95 |
| GoF | 1.2 |
| Reduced χ^2^ | 1.39 |

Table 2: Occupied Wyckoff sites, refined atomic coordinates (Å) of EuSiN_2_. (estimated standard deviations in parentheses).

| **Compound** | **Atom** | **Wyck.** | ***x*** | ***y*** | ***z*** | **Occ.** | **U_iso_** |
| --- | --- | --- | --- | --- | --- | --- | --- |
| **EuSiN_2_** | **Eu1** | 4*e* | 0.34083(13) | 0.57128(7) | 0.17583(15) | 1 | 0.0144(2) |
|  | **Si1** | 4*e* | 0.1088(4) | 0.1458(2) | 0.0653(5) | 1 | 0.0144(2) |
|  | **N1** | 4*e* | 0.2303(7) | 0.5917(7) | 0.5962(9) | 1 | 0.0144(2) |
|  | **N2** | 4*e* | 0.2172(7) | 0.2353(6) | 0.3801(11) | 1 | 0.0144(2) |

Table 3: Selected bond lengths (Å) of EuSiN_2_. (standard deviations in parentheses).

| **Atom** | **Distance** | **Atom** | **Distance** |
| --- | --- | --- | --- |
| Eu1-N1 | 2.643(5) | Eu1-N1 | 2.528(5) |
| Eu1-N1 | 2.642(4) | Eu1-N2 | 2.682(4) |
| Eu1-N1 | 2.901(5) | Eu1-N2 | 3.018(5) |
| Eu1-N1 | 2.988(5) | Eu1-N2 | 3.127(4) |
|  |  |  |  |
| Si1-N1 | 1.853(5) | Si1-N2 | 1.649(6) |
| Si1-N1 | 1.901(4) | Si1-N2 | 1.716(6) |


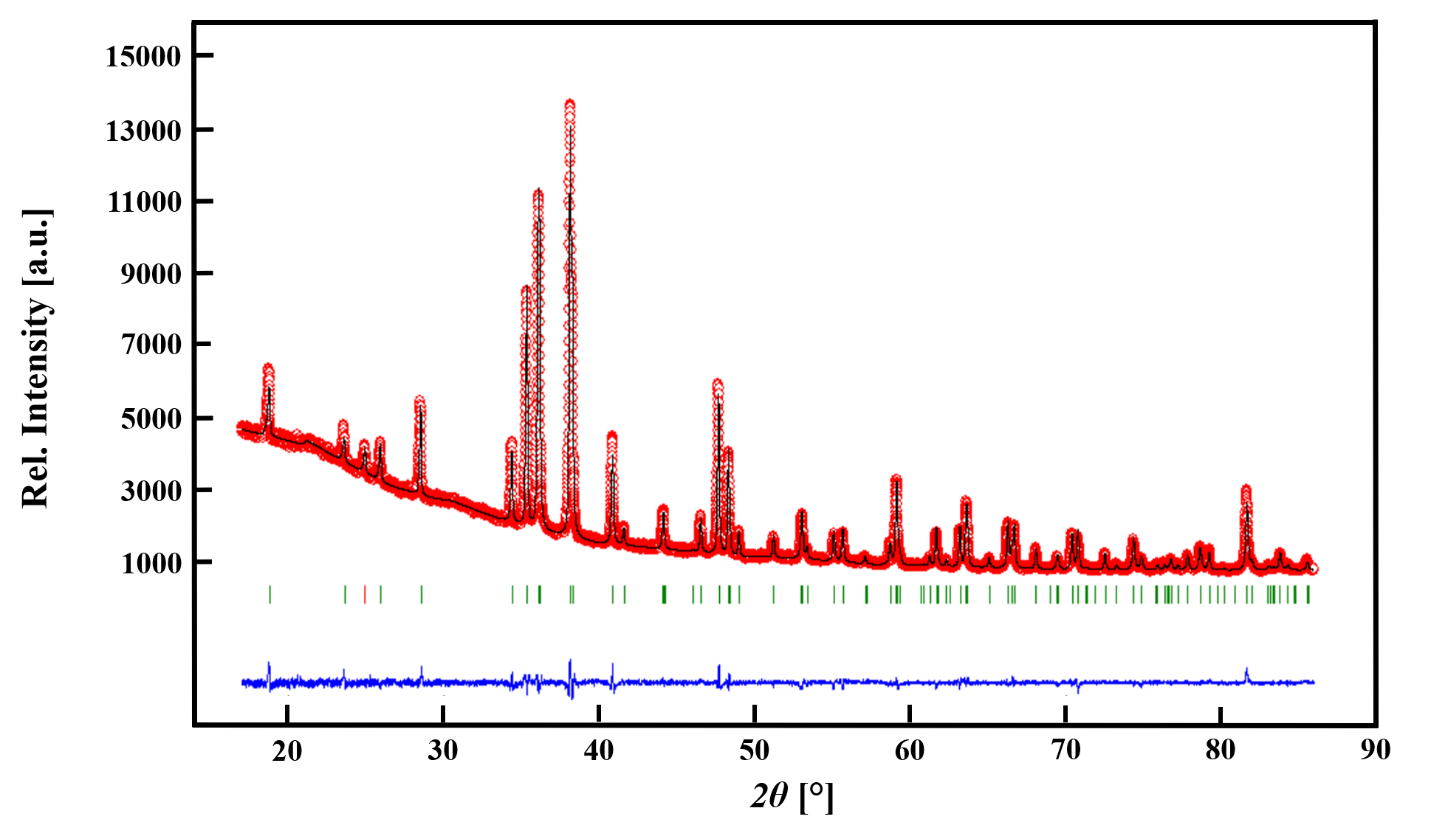


Figure 1: Observed (black) and calculated (red) X-ray powder diffraction pattern of EuSiN_2_ as well as the position of Bragg reflections (green bars) together with their difference curve (blue) after Rietveld refinement (λ = 1.788965 Å).

EuSiN_2_, being isotypical to SrSiN_2_, crystallizes in space group *P*2_1_*/c* and is built up by a two-dimensional highly condensed nitridosilicate framework with vertex- and edge-sharing SiN_4_-tetrahedra (see Figure 2a). The structure consists of pairs of edge-sharing SiN_4_-tetrahedra to form bow-tie-shaped Si_2_N_6_ dimers, which share vertexes to form layers (see Figure 3a and Figure 4a).

In EuSiN_2_ these SiN_4_-tetrahedra layers were separated by cations (see Figure 3a) whereas taking other alkaline-earth elements into account, cation size plays a dominant role for the structure formation. ^[10,29]^

This structure motif can be found for *M*SiN_2_ (*M* = Sr, Ba)^[10,29]^ as well (Figure 3b, d), while CaSiN_2_ has a SiN_4_-tetrahedra network structure. (see Figure 2c, Figure 3c) But it has to be mentioned that Sr- and BaSiN_2_^[10][29]^ are not isotypical, what becomes clear having a look at Figure 2b, d and Figure 4b, c. In EuSiN_2_, having only one crystallographic *M* cation site, an 8-fold nitrogen coordination is observed as it can be found in *M*SiN_2_ (*M* = Sr, Ba)^[10,29]^ as well (see Figure 4d).

In CaSiN_2_,^[10,29]^ being isotypical to KGaO_2_^[56]^ as well as HP-CaSiN_2,_^[13]^ these SiN_4_-tetrahedra build condensed *Sechser*-rings^[57]^ with two different formations (see Figure 2c). For EuSiN_2_ and SrSiN_2_ the triangle formations have disappeared and only distorted *Sechser*-rings^[57]^ remain (see Figure 2a, b). The endmember BaSiN_2_ however displays with 119° nearly perfectly shaped *Sechser*-rings^[57]^ (see Figure 2d) So it becomes clear that the cation size plays a very important role for the crystal structure as was already shown for other nitridosilicates.^[58]^

SrSiN_2_^[10,29]^ crystallizes in the same space group as EuSiN_2_ (*P*2_1_/*c*) to which it is isotypical and has only very slight differences in distances and angles, due to the very similar ionic radii of Eu^2+^ (1.17 Å) and Sr^2+^ (1.18 Å) (see Figure 2b and Figure 4a, b).

The Eu-N and Sr-N polyhedra, as shown in Figure 4a, b, e, f, display the same bow-tie-shaped structure motif resembling those of the edge sharing SiN_4_-tetrahedra.

The bow-tie formations of the Eu-N polyhedra exhibit two different Eu-Eu distances, one for in between the edge sharing Eu-N polyhedra (3.46 Å) and one for two neighbouring Eu-N polyhedra (3.36 Å) (see Figure 4a). This feature can be found for the Sr-N polyhedra as well (see Figure 4b).

Even if there is an 8-fold nitrogen coordination of the cations, (see Figure 4d) the bow-tie formation of the Eu-N and Sr-N polyhedra has been choosen as it enables an explicit perspective of the layer formation.

The distances between the layers differ only marginally for Eu-Eu (3.46 Å, 3.36 Å) and Sr-Sr (3.47 Å, 3.38 Å). (see Figure 4a, b), as well as the interlayer distances (EuSiN_2_ : 4.04 Å and SrSiN_2_ 4.03 Å).^[10,29]^

BaSiN_2_^[10,29]^ differs not only from the perfect hexagonal formation of the SiN_4_-tetrahedra (see Figure 2d), but also features pentagonal pyramidal Ba-N polyhedra, forming double layers being shifted by one half (see Figure 4c, g). The distance between two Ba atoms is with 3.96 Å much larger than for the neighbouring Eu- and Sr-atoms in *M*SiN_2_ (*M* = Eu, Sr) (see Figure 4a, b), reflecting the decisive influence of the cation. CaSiN_2_ has not been depicted in Figure 4, as there is no layer formation of the Ca-N polyhedra in CaSiN_2_.


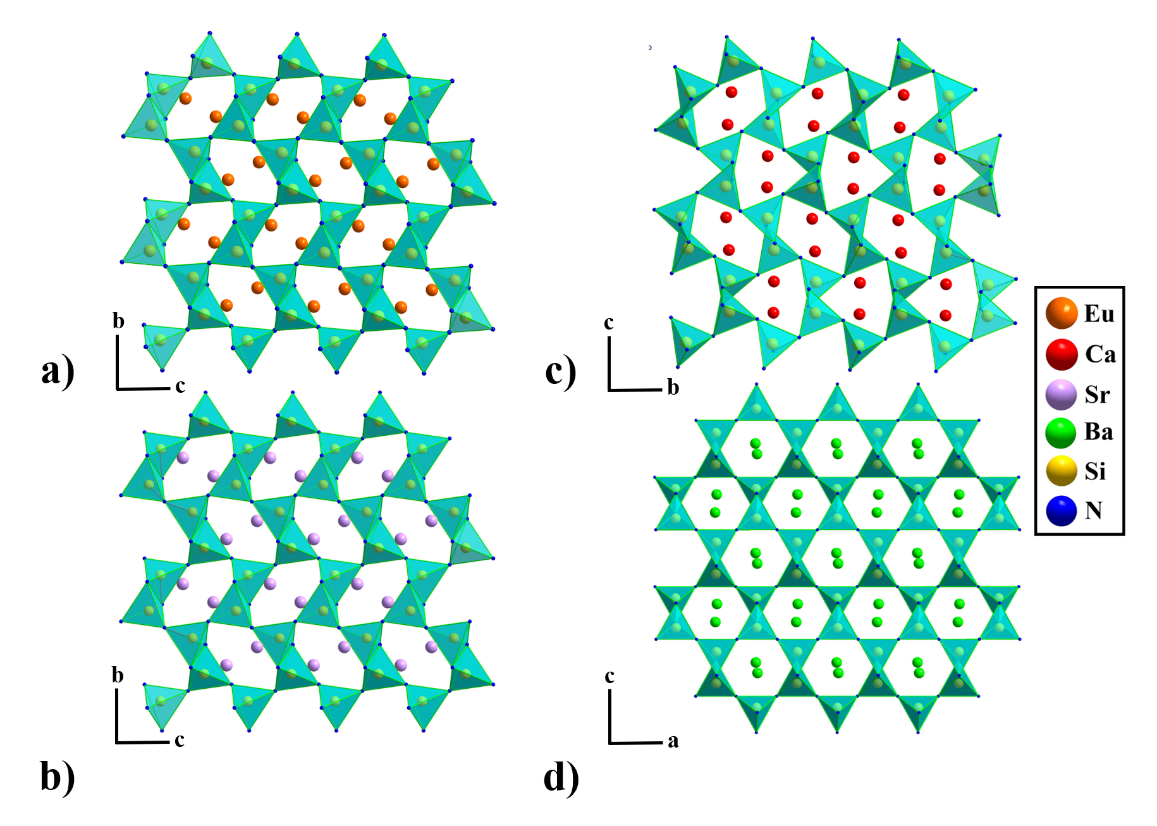


Figure 2: a) EuSiN_2_ along the [100] direction, b) SrSiN_2_^[10,29]^ along the [100] direction, c) CaSiN_2_ ^[10,12]^ along the [100] direction and d) BaSiN_2_^[10,29]^ along the [010] direction. (Eu atoms are depicted in orange, Ca in red, Sr in violet, Ba in green, Si in yellow and N in blue).


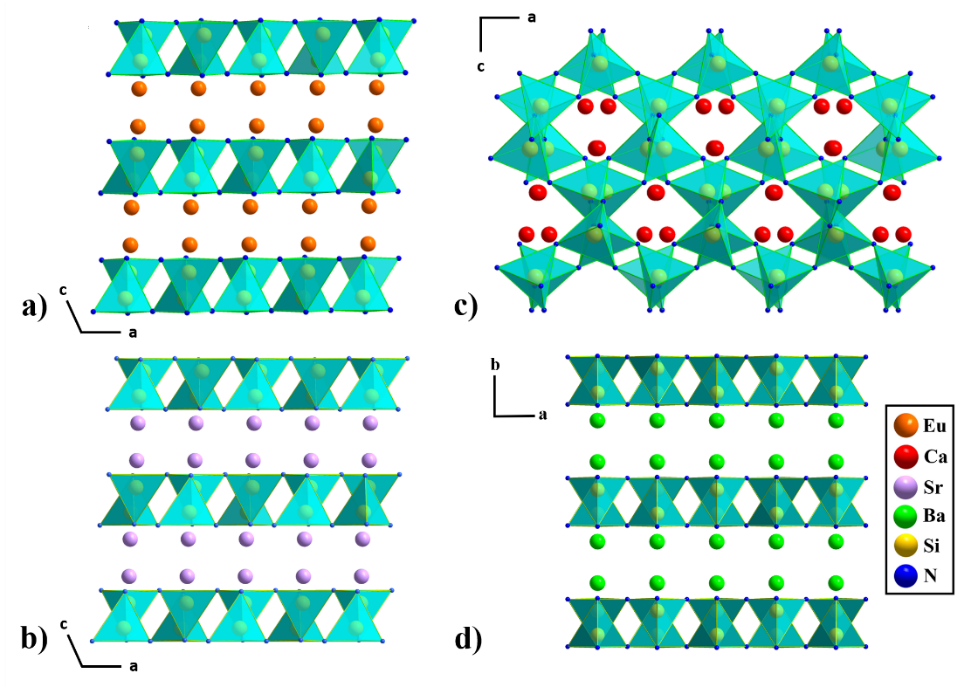


Figure 3: a) EuSiN_2_ in the [010] direction, b) SrSiN_2_^[10,29]^ in the [010] direction, c) CaSiN_2_^[10,12]^ in the [010] direction and d) BaSiN_2_^[10,29]^ in the [001] direction. (Eu atoms are depicted in orange, Ca in red, Sr in violet, Ba in green, Si in yellow and N in blue.)


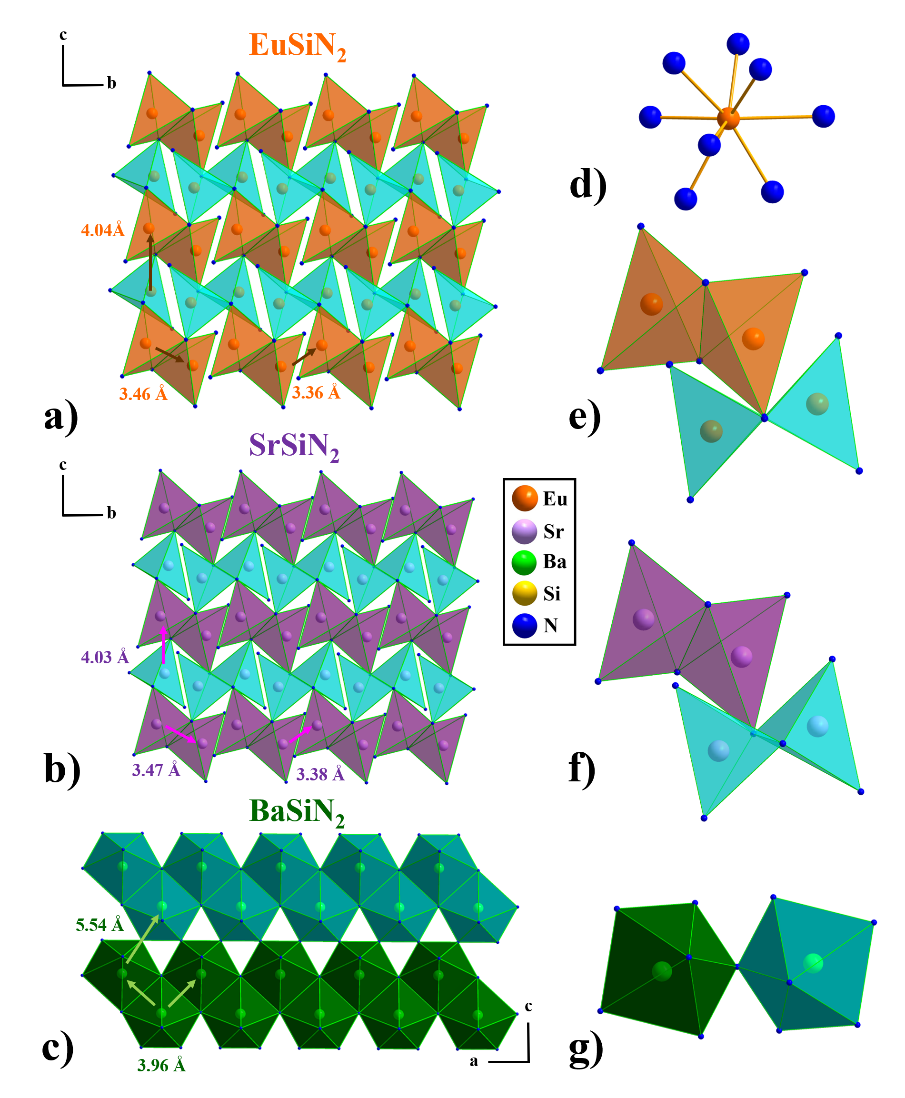


Figure 4: *M*-N polyhedra layers in a) EuSiN_2_ in the quasi [100] direction (layers in orange and cyan), b) SrSiN_2_^[10,29]^ in the quasi [100] direction (layers in violet and cyan), and c) BaSiN_2_^[10,29]^ in the [010] direction (layers in dark green and petrol), d) 8-fold nitrogen coordination of the Eu atom, e) bow-tie formation of the Eu-N polyhedra, f) bow-tie formation of the Sr-N polyhedra and g) pentagonal pyramidal formation of the Ba-N polyhedra. (Eu atoms are depicted in orange, Sr in violet, Ba in green, Si in yellow and N in blue.)

***Luminescence properties***

The emission and absorption spectra of Eu^2+^ usually consist of broad bands due to transitions between the ^8^S_7/2_ (4f^7^) ground state and the crystal field components of the 4f^6^5d excited state configuration.^[59]^ The characteristics of the f-d luminescence of divalent europium (4f^7^) in different compositions has been subject of many investigations.^[60–62]^

The excitation (399 nm, 421nm, 441nm, 451nm, 469nm, 483nm, 493nm) and emission (708 nm, 764 nm) spectra of EuSiN_2_ are presented in Figure 5a.

They have been recorded two times, once with the excitation wavelength λ_ex._ = 365 nm and for the second time with λ_ex._ = 469 nm to be sure that the spectra are no artefact and reproducible. The structure in the excitation band of EuSiN_2_ (see Figure 5a left side) can be ascribed to 4f^7^ to 4f^6^5d^1^ transition of the Eu^2+^ ion on the single *M* site. The energy of the Eu^2+^ 4f-5d excitation is strongly dependent on the local environment because of the local coordination and the crystal field splitting of the 5d levels.^[63]^

If the crystal field is weak and the amount of covalency low, the 4f^6^5d configuration of the Eu^2+^ ion shifts to higher energies and lies therefore above the ^6^P_7/2_ level which results at low temperatures in a sharp line emission due to the ^6^P_7/2_ to ^8^S_7/2_ transition, as it is e.g. the case for SrB_4_O_7_:Eu^2+^.^[64,65]^ And Figure 5a shows this phenomenon of sharp line emission, which has already been observed several times in literature ^[29,66–70]^ for EuSiN_2_ quite well (see Figure 6, Figure 7 and Figure 9b).

For a comparison the excitation spectra of SrSiN_2_:Eu^2+^ and BaSiN_2_:Eu^2+[29]^ are depicted in Figure 6a and b. Here, especially for BaSiN_2_:Eu^2+^,^[29]^ only three dominant peaks are visible. Duan *et al.* ^[29]^ claim these peaks are due to the splitting of the 5d bands and because of an overlap only three dominating 5d bands can be observed in the excitation spectra of Eu^2+^-doped *M*SiN_2_ (*M* = Sr, Ba).^[29]^

While Poort *et al.*^[66]^ had to perform their measurements of SrSiO_3_:0.5% Eu^2+^ in 1996 at 4K (see Figure 7a) to get a detailed resolution of the fine structure, we get nowadays an excellent resolution and reliable results with the state-of-the-art photoluminescence spectrometer a room temperature as well^[69]^ (see Figure 5a left side and Figure 7b).

In the CIE 1931 diagram, (see Figure 5b) the chromaticity coordinate positions of EuSiN_2_ (*x* = 0.678, *y* = 0.284) are indicated with a black circle, which corresponds to an intensive red.


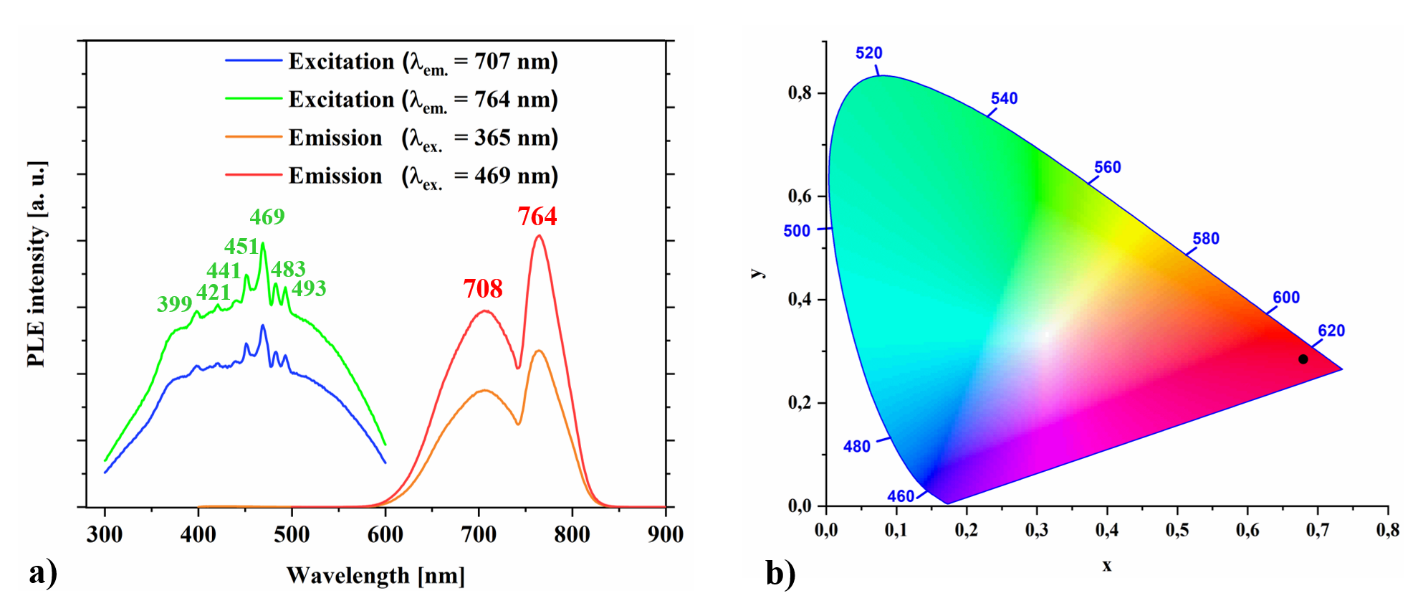


Figure 5: a) Excitation (green and blue) and emission spectra (red and orange) of EuSiN_2_, b) CIE 1931 diagram of EuSiN_2_.


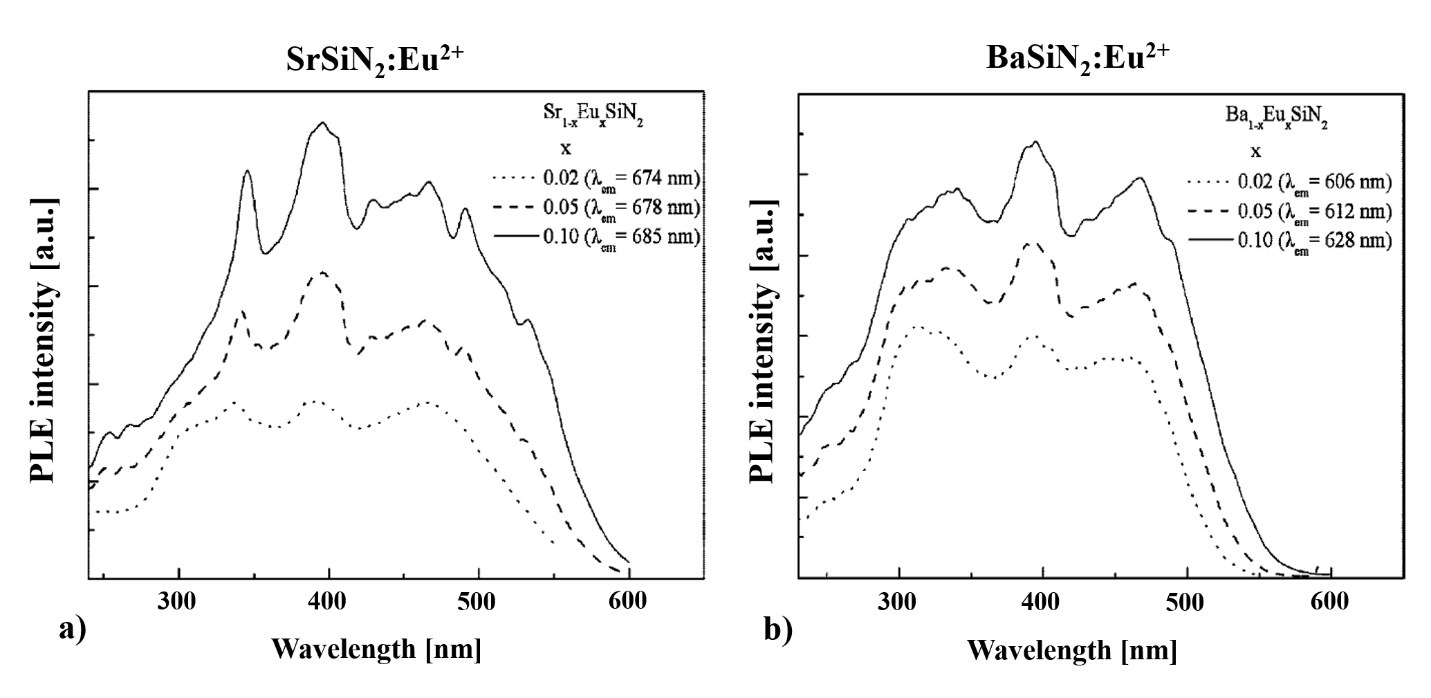


Figure 6: Excitation spectra a) SrSiN_2_:Eu^2+^ and b) BaSiN_2_:Eu^2+^.^[29]^


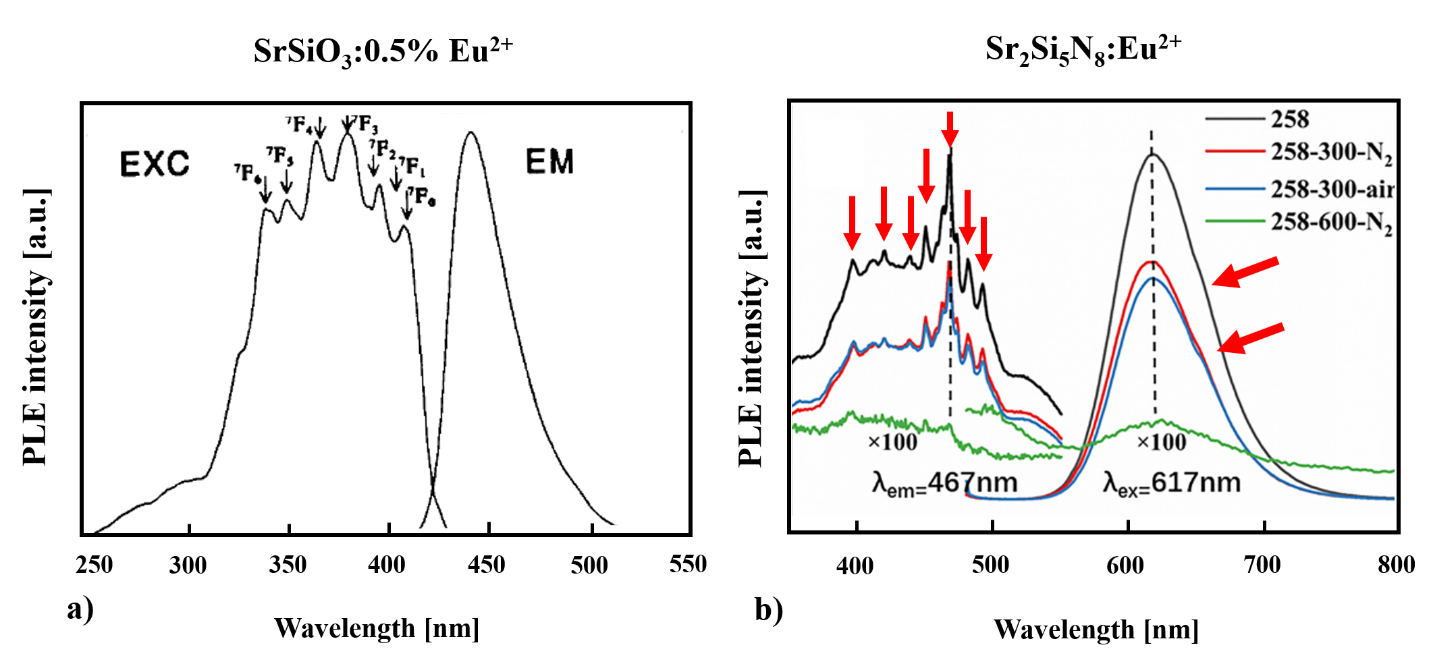


Figure 7: Excitation and emission spectra of a) SrSiO_3_:0.5% Eu^2+[66]^ and b) Sr_2_Si_5_N_8_:Eu^2+^.^[69]^


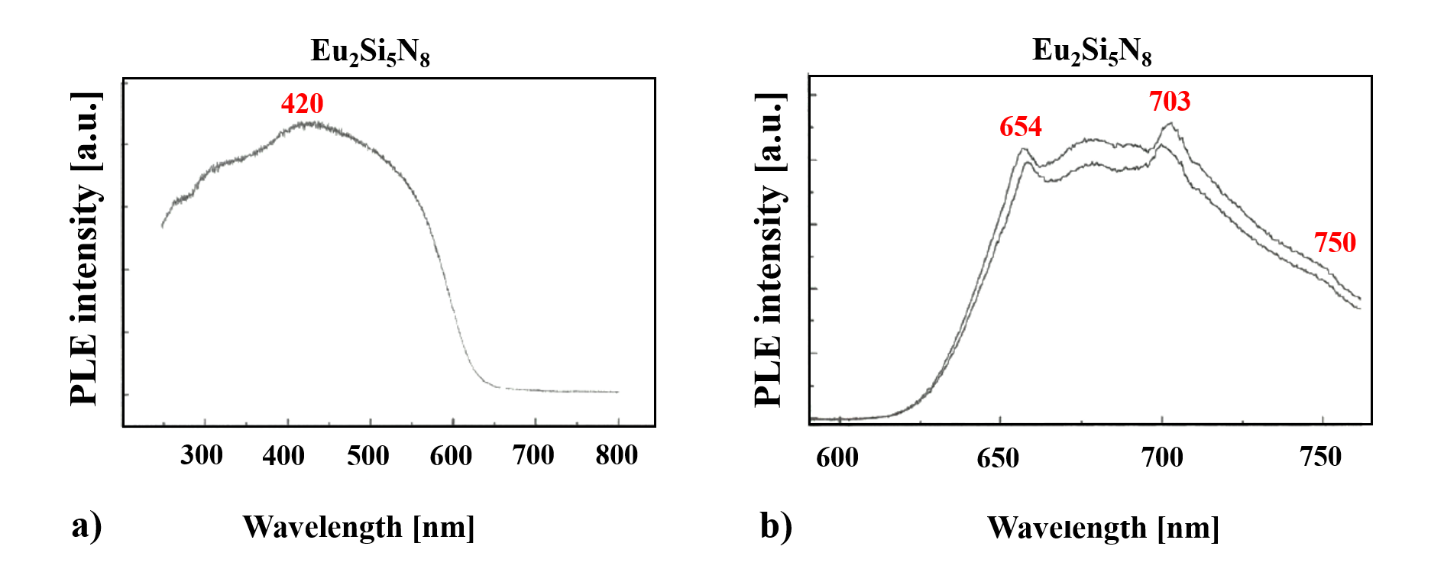


Figure 8: Absorption a) and emission spectra b) of Eu_2_Si_5_N_8_^[34,53]^

The emission spectrum of EuSiN_2_ consists of two luminescence peaks, one at 708 nm and the other one at 764 nm (see Figure 5a right side). Having a look into literature we see many compounds as e.g. Sr_2_Si_5_N_8_:Eu^2+[69,71]^, Sr_2–_*_x_*La*_x_*Si_5–_*_x_*Al*_x_*N_8_:Eu^2+[72]^ and Ba_1.89_Eu_0.11_Si_5_N_8_.^[70]^ showing more or less developed shoulders at their luminescence peaks , but observing two fully developed emission peaks is a novel feature for nitridosilicates (see Figure 7b, Figure 9a,b). For Ba_0.99_Eu_0.01_Si_2_O_2_N_2_^[73]^ having only one Ba site however the two luminescence peaks are more distinct.

The 5d^1^ orbitals are much more sensitive to the local surrounding than the 4f orbitals. Possible defects or vacancies in the EuSiN_2_ structure could present a different crystallographic surrounding, strongly reflecting the energy position of the 5d orbitals. Such possibility would give rise to two luminescence peaks.

Another way of classifying this phenomenon is to look at the family compound Eu_2_Si_5_N_8_^[34,51–53]^ and the luminescence spectra Huppertz displayed in his dissertation.^[53]^ Here we see clearly that already for Eu_2_Si_5_N_8_^[34,53]^ (see Figure 8b) there are two luminescent peaks, one at 654 nm and the other one at 703 nm. At 750 nm an additional shoulder becomes visible.

Huppertz^[53]^ explains the luminescence peaks of Eu_2_Si_5_N_8_^[34][53]^ due to an two-photon absorption of the europium ion. This spectrum was produced by a high intensity laser with an excitation wavelength of 523.5 nm.

Nonlinear optical behaviour investigations (NLO)^[74]^ have been performed for many nitridosilicates including as well Eu_2_Si_5_N_8_.^[34,51–53]^ In the course of this investigations also the two-photon absorption of the Eu^2+^ ion in nitridosilicates played an important role. ^[34,51–53][70]^

Two-photon absorption, being the simultaneous [absorption](https://en.wikipedia.org/wiki/Absorption_(electromagnetic_radiation)) of two [photons](https://en.wikipedia.org/wiki/Photon) of identical or different frequencies in order to [excite](https://en.wikipedia.org/wiki/Electron_excitation) an [atom](https://en.wikipedia.org/wiki/Atom) from the [ground state](https://en.wikipedia.org/wiki/Ground_state) via a virtual energy level to a higher [excited electronic state](https://en.wikipedia.org/wiki/Excited_electronic_state), was originally predicted by Maria Goeppert-Mayer in 1931 in her dissertation^[75]^ and in 1961 the first experimental verification of the two-photon excitation was found in CaF_2_: Eu^2+^.^[76]^

Ba_2-x_Eu_x_Si_5_N_8_,^[70]^ revealing long lasting luminescence and thermoluminescence shows very broad absorption and emission bands being typical for Eu^2+^. The two emission bands at 610 nm and 630 nm are broadened due to the presence of two overlaying crystallographic Eu^2+^ sites. Höppe *et al.*^[70]^ believed these two neighbouring wavelengths around 600 nm should be mainly due to an efficient two-photon absorption. The exceptional strong red shift is ascribed to the different nitrogen coordination of the Eu^2+^ ions^[51][70]^ (see Figure 9b, and Figure S1).


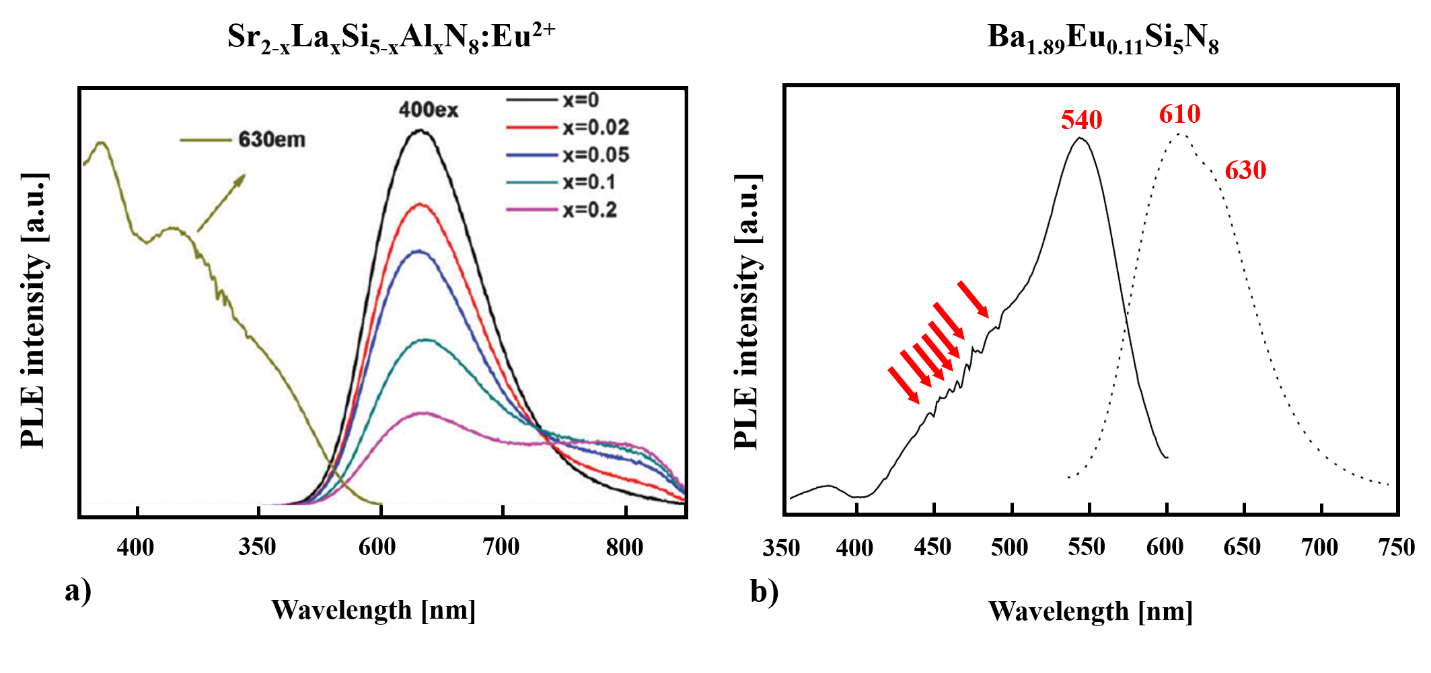


Figure 9: a) Excitation and emission spectra of Sr_2–_*_x_*La*_x_*Si_5–_*_x_*Al*_x_*N_8_:Eu^2+ [72]^ and b) Ba_1.89_Eu_0.11_Si_5_N_8_.^[70]^

In Sr_2_Si_5_N_8_^[72]^ there are only two types of Sr^2+^ sites for Eu^2+^ substitution, but Figure 9a and Figure S2b show that there are three Gaussian peaks fitting the emission band. Chuang and coworkers^[72]^ assume that the incorporation of La^3+^- Al^3+^ ions leads to an additional site occupied by Eu^2+^, not changing the Sr_2_Si_5_N_8_ macrostructure but only modifying the micro-structure around Eu^2+^ in the host lattice. But they do not take into account a possible two-photon absorption for their explanation of the three luminescent peaks. Here as well unusual long-wavelength excitation and emission bands are observed which result from the nitrogen-rich surrounding of the Eu^2+^ ions in Sr_2_Si_5_N_8_.

As for the two compounds^[51,72]^ mentioned above, we see for EuSiN_2_ a very strong red shift

as well. With an 8-fold nitrogen coordination of the cations in EuSiN_2_, like in SrSiN_2_:Eu^2+^,^[29]^ we have as well a nitrogen-rich surrounding of the Eu^2+^ ions.


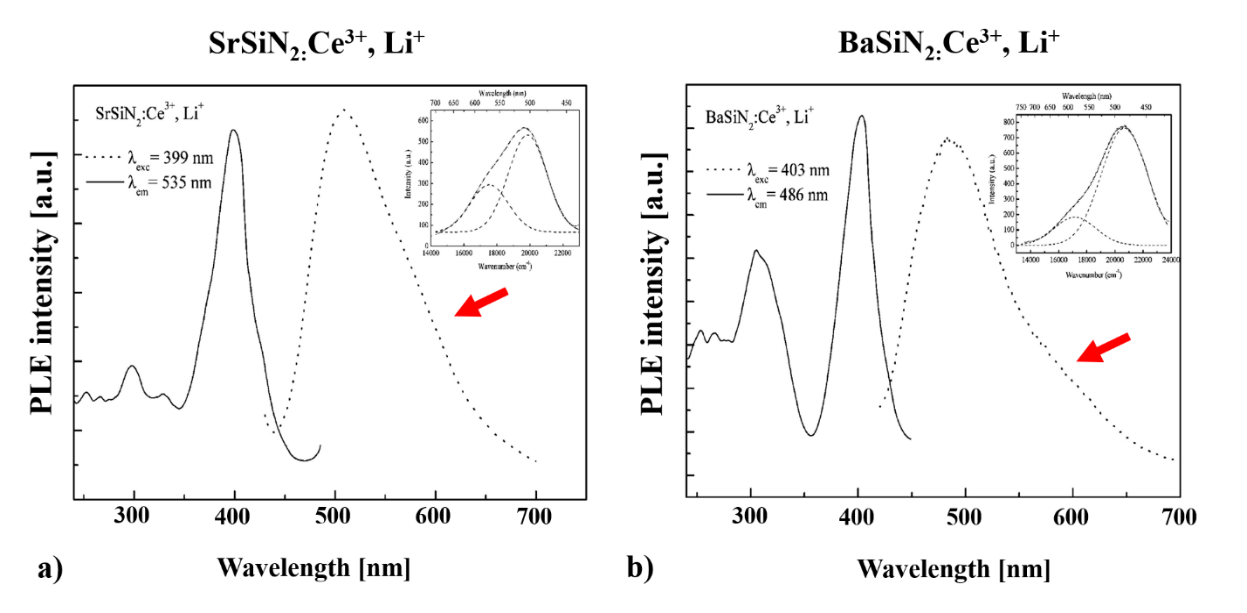


Figure 10: Excitation and emission spectra a) SrSiN_2_:Ce^3+^, Li^+[29]^ and b) BaSiN_2_:Ce^3+^, Li^+^.^[29]^

For SrSiN_2_:Ce^3+^, Li^+^ as well as BaSiN_2_:Ce^3+^, Li^+[29]^ a shoulder in their emission spectra appears as well, (see Figure 10) but two fully generated luminescence peaks are only distinctive for EuSiN_2_. The inserted pictures in Figure 10 show that these peaks can be fitted very well with two Gaussian functions, which proves the presence of the shoulder. However Duan *et al.*^[29]^ provide no explanation for this feature. For the Eu-doped compounds, SrSiN_2_:Eu^2+[29]^ and BaSiN_2_:Eu^2+^,^[29]^ no shoulder can be detected in the emission spectrum, only for Ba_1–_*_x_*Eu*_x_*SiN_2_:Eu^2+[29]^ (*x* = 0.1) a very first sign of a possible shoulder is implied (see Figure S3).

***Conclusions***

With EuSiN_2_ a novel Eu-nitridosilicate is presented including a very comprehensive structure description and a comparison with its related compounds *M*SiN_2_ (*M* = Ca, Sr, Ba). A different way of presenting the structure as *M*-N polyhedra layers is introduced. Furthermore, the luminescence characteristics of EuSiN_2_ are studied.

The phenomenon of sharp peaks in the excitation band of EuSiN_2_ can be ascribed to 4f^7^ to 4f^6^5d^1^ transition of the Eu^2+^ ion and has already been observed several times in literature. In former times it was necessary to cool down the sample to very low temperatures to get a detailed structure of the excitation bands. Nowadays state-of-art photoluminescence spectrometer provide an excellent resolution and reliable results a room temperature as well.

The emission spectrum of EuSiN_2_ consists of two luminescence peaks, one at 708 nm and the other one at 764 nm.

Having a look into literature many europium doped nitridosilicates exhibit more or less developed shoulders for their luminescence peaks, but two clearly shaped emission peaks are a novel feature for nitridosilicates.

For the family compound Eu_2_Si_5_N_8_ two luminescent peaks can be found as well, and Huppertz explained them by a two-photon absorption of the Eu^2+^ ion. Also Ba_1.98_Eu_0.11_Si_5_N_8_ shows this phenomenon and Höppe argues with an efficient two-photon absorption too. Höppe also proposes that the two luminescent peaks could be caused by two overlaying crystallographic Eu sites. EuSiN_2_ has only one crystallographic site, which makes the two-photon absorption more probable.

But we cannot rule out that possible defects and vacancies could influence the 5d^1^ orbitals presenting a different crystallographic surrounding for the Eu ion and giving rise to the two luminescence peaks.

Therefore we would like to concur with the explanation of Huppertz and Höppe and propose likewise a two-photon absorption for EuSiN_2_ being the reason for the two dinstinct luminescence peaks in the emission spectrum.

But to be absolutely sure that the high intense laser light is needed and the two-photon absorption could not perhaps take place with excitation light of lower intensity as well this will be part of a future project.

This luminescence feature is not only present for the 2-5-8 phases, but already visible for Sr- and BaSiN_2_:Ce^3+^,Li^+^ as well, having a shoulder at their luminescence peaks.

As alkaline-earth silicon nitrides MSiN_2_ (M = Sr, Ba) have been already several times investigated because of their electronic properties, especially their band gap features e.g as semiconductors, it would be very interesting to investigate the nonlinear optical behavior of EuSiN_2_ in a future project, just as the magnetism of the Europium will be a subject of further studies.

***Experimental Section***

All sample handlings were performed in inert atmosphere using an argon-filled glove box (MBraun Labmaster, ρ(O_2_) / ρo < 0.1 ppm, ρ (H_2_O) / ρo < 0.1 ppm) due to the extreme sensitivity of both the starting materials and some of the final products to moisture and air.

**Synthesis of EuN:**

For the synthesis of EuN, Eu (Hunan Rare Earth Metals Materials Co, 99.9%) was put in a tungsten crucible, placed into a water-cooled quartz reactor and mounted into a radio-frequency furnace^[77]^ under flowing N_2_ atmosphere (Nippon Gases 99.999%, purified with an oxysorb cartridge (Messer Griesheim)) of ambient pressure for 30 min at maximally 850 °C. Great care has been taken, that the Eu does not melt prior or during the early stages of the reaction. After the reaction, the sample was finely ground in an agate mortar and sieved (100 mesh).

**Synthesis of EuSiN_2_:**

For the synthesis of EuSiN_2_ a mixture of 433.27 mg EuN and 69.37 mg Si_3_N_4_ (β-Si_3_N_4_, Chempur 99.999%) were mixed in an agate mortar and pressed into a pellet with 8 or 10 mm diameter.

The pellet was placed into a tantalum crucible under argon atmosphere in a glovebox and the set up was placed into a water-cooled quartz reactor of a radio-frequency furnace^[77]^ and hold at 1550 °C for 20 minutes under nitrogen atmosphere (Eq. (1)). By this reaction a dark red powder was obtained.

***X-ray diffraction***

X-ray diffraction experiments on EuSiN_2_ powder samples were performed on a STOE STADI P powder diffractometer in Debye-Scherrer geometry with Ge (111)-monochromatized Co-*Kα*_1_ radiation (*λ* = 1.788965 Å). The samples were enclosed in a glass capillary of 0.3 mm diameter.

The deposition number CSD-2435060 for EuSiN_2_ (<https://www.ccdc.cam.ac.uk/services/structures?id=doi:10.1002/chem.202404395>) contains the supplementary crystallographic data for this paper. These data are provided free of charge by the joint Cambridge Crystallographic Data Centre and Fachinformationszentrum Karlsruhe (<http://www.ccdc.cam.ac.uk/structures>).

***Luminescence***

Steady-state emission and excitation spectra presented in this study have been recorded using the Fluorolog-QM photoluminescence (PL) spectrometer from HORIBA scientific working with a continuous 75 W Xe lamp and a cooled Hamamatsu R928P photomultiplier in TE-cooled housing. The emission and excitation spectra were corrected for the instrumental artefacts using the supplied correction factors. A 400 nm long pass filter was used to eliminate the second order artefacts.

***Acknowledgement***

The authors gratefully acknowledge Dr. Juri Prots, Liuda and Valeriu Mereacre for helpful discussions.

Structure and Photoluminescence Properties of EuSiN2 © 2023 by Cordula Braun is licensed under CC BY-SA 4.0. To view a copy of this license, visit http://creativecommons.org/licenses/by-sa/4.0/

***References***

[1] P. Ball, *Nat. Mater.* **2015**, *14*, 453–453.

[2] P. Pust, P. J. Schmidt, W. Schnick, *Nat. Mater.* **2015**, *14*, 454–458.

[3] P. Bielec, W. Schnick, *Angew. Chemie* **2017**, *129*, 4888–4891.

[4] P. Bielec, O. Janka, T. Block, R. Pöttgen, W. Schnick, *Angew. Chemie Int. Ed.* **2018**, *75*, 2409–2412.

[5] P. Bielec, R. Nelson, R. P. Stoffel, L. Eisenburger, D. Günther, A.-K. Henss, J. P. Wright, O. Oeckler, R. Dronskowski, W. Schnick, *Angew. Chemie* **2018**, *58*, 1432–1436.

[6] A. Rabenau, P. Eckerlin, *Naturwissenschaften* **1959**, *46*, 106–107.

[7] H. T. Hintzen, R. J. Bruls, A. C. A. Delsing, K. Itatani, S. Tanaka, G. de With, R. Metselaar, *Key Eng. Mater.* **2002**, *206*–*213*, 973–976.

[8] R. J. Bruls, H. T. Hintzen, R. Metselaar, *J. Eur. Ceram. Soc.* **2005**, *25*, 767–779.

[9] W. A. Groen, M. J. Kraan, G. de With, *J. Eur. Ceram. Soc.* **1993**, *12*, 413–420.

[10] Z. A. Gál, P. M. Mallinson, H. J. Orchard, S. J. Clarke, *Inorg. Chem.* **2004**, *43*, 3998–4006.

[11] T. de Boer, J. Häusler, P. Strobel, T. D. Boyko, S. S. Rudel, W. Schnick, A. Moewes, *J. Phys. Chem. C* **2021**, *125*, 27959–27965.

[12] F. Ottinger, R. Nesper, *Acta Crystallogr. A* **2002**, *58*, c337–c337.

[13] C. Braun, H. Ehrenberg, W. Schnick, *Eur. J. Inorg. Chem.* **2018**, *2018*, 1107–1113.

[14] Y. Laurent, *Rev. Chim. Miner.* **1968**, *5*, 1019–1050.

[15] W. A. Groen, M. J. Kraan, G. De With, *J. Mater. Sci.* **1994**, *29*, 3161–3166.

[16] R. J. Bruls, H. T. Hintzen, R. Metselaar, *J. Mater. Sci.* **1999**, *34*, 4519–4531.

[17] P. E. D. Morgan, *J. Mater. Sci. Lett.* **1984**, *3*, 131–132.

[18] R. J. Bruls, A. A. Kudyba-Jansen, P. Gerharts, H. T. Hintzen, R. Metselaar, *J. Mater. Sci. Mater. Electron.* **2002**, *13*, 63–75.

[19] H. Hayashi, K. Hirao, M. Toriyama, S. Kanzaki, K. Itatani, *J. Am. Ceram. Soc.* **2001**, *84*, 3060–3062.

[20] M. Zeuner, S. Pagano, W. Schnick, *Angew. Chem. Int. Ed. Engl.* **2011**, *50*, 7754–7775.

[21] H. Lange, G. Wötting, G. Winter, *Angew. Chem. Int. Ed. Engl.* **1991**, *30*, 1579–1597.

[22] Y.-C. Chiu, C.-H. Huang, T.-J. Lee, W.-R. Liu, Y.-T. Yeh, S.-M. Jang, R.-S. Liu, *Opt. Express* **2011**, *19*, A331.

[23] C. Kulshreshtha, J. H. Kwak, Y.-J. Park, K.-S. Sohn, *Opt. Lett.* **2009**, *34*, 794–796.

[24] R. Le Toquin, A. K. Cheetham, *Chem. Phys. Lett.* **2006**, *423*, 352–356.

[25] S. S. Lee, S. Lim, S.-S. Sun, J. F. Wager, in (Eds.: A. Hariz, V.K. Varadan, O. Reinhold), International Society For Optics And Photonics, **1997**, pp. 75–83.

[26] X.-M. Wang, X. Zhang, S. Ye, X.-P. Jing, *Dalt. Trans.* **2013**, *42*, 5167–5173.

[27] Y. Q. Li, N. Hirosaki, R.-J. Xie, T. Takada, Y. Yamamoto, M. Mitomo, K. Shioi, *Int. J. Appl. Ceram. Technol.* **2010**, *7*, 787–802.

[28] T. De Boer, T. D. Boyko, C. Braun, W. Schnick, A. Moewes, *Int. J. Appl. Ceram. Technol.* **2022**, 1–7.

[29] C. J. Duan, X. J. Wang, W. M. Otten, A. C. A. Delsing, J. T. Zhao, H. T. Hintzen, *Chem. Mater.* **2008**, *20*, 1597–1605.

[30] X.-H. He, N. Lian, J.-H. Sun, M.-Y. Guan, *J. Mater. Sci.* **2009**, *44*, 4763–4775.

[31] R. J. Xie, N. Hirosaki, *Sci. Technol. Adv. Mater.* **2007**, *8*, 588–600.

[32] K. Uheda, N. Hirosaki, H. Yamamoto, *Phys. Status Solidi A* **2006**, *203*, 2712–2717.

[33] C. Braun, Neue Hochdruckphasen der Nitrido- und Oxonitridosilicate und verwandter binärer Nitride, doctoral thesis, Ludwig-Maximilians-Universität München, **2010**.

[34] H. Huppertz, W. Schnick, *Acta Crystallogr. C* **1997**, *53*, 1751–1753.

[35] Y. Q. Li, C. M. Fang, G. De With, H. T. Hintzen, *J. Solid State Chem.* **2004**, *177*, 4687–4694.

[36] M. Zeuner, S. Pagano, P. Matthes, D. Bichler, D. Johrendt, T. Harmening, R. Pöttgen, W. Schnick, *J. Am. Chem. Soc.* **2009**, *131*, 11242–11248.

[37] S. R. Römer, C. Braun, O. Oeckler, P. J. Schmidt, P. Kroll, W. Schnick, *Chem. Eur. J.* **2008**, *14*, 7892–7902.

[38] C. Braun, M. Seibald, S. L. Börger, O. Oeckler, T. D. Boyko, A. Moewes, G. Miehe, A. Tücks, W. Schnick, *Chem. - A Eur. J.* **2010**, *16*, 9646–9657.

[39] C. Braun, S. L. Börger, T. D. Boyko, G. Miehe, H. Ehrenberg, P. Höhn, A. Moewes, W. Schnick, *J. Am. Chem. Soc.* **2011**, *133*, 4307–4315.

[40] C. Braun, H. Ehrenberg, W. Schnick, *Eur. J. Inorg. Chem.* **2012**, *2012*, 3923–3928.

[41] R. Mueller-Mach, G. Mueller, M. R. Krames, H. A. Höppe, F. Stadler, W. Schnick, T. Juestel, P. Schmidt, *Phys. Status Solidi A* **2005**, *202*, 1727–1732.

[42] Y. Q. Li, G. de With, H. T. Hintzen, *J. Mater. Chem.* **2005**, *15*, 4492–4496.

[43] X. Piao, T. Horikawa, H. Hanzawa, K. Machida, *Appl. Phys. Lett.* **2006**, *88*, 161908-1-161908–3.

[44] R. J. Xie, N. Hirosaki, Y. Li, T. Takeda, *Materials (Basel).* **2010**, *3*, 3777–3793.

[45] R. Mueller-Mach, G. O. Mueller, M. R. Krames, O. B. Shchekin, P. J. Schmidt, H. Bechtel, C. H. Chen, O. Steigelmann, *Phys. Status Solidi - Rapid Res. Lett.* **2009**, *3*, 215–217.

[46] T. Jüstel, H. Nikol, C. Ronda, *Angew. Chem. Int. Ed. Engl.* **1998**, *37*, 3084–3103.

[47] C. Ronda, Ed. , *Luminescence*, Wiley-VCH Verlag GmbH & Co. KGaA, Weinheim, Germany, **2007**.

[48] W. Schnick, *Phys. Status Solidi - Rapid Res. Lett.* **2009**, *3*, 1–2.

[49] P. Schmidt, A. Tuecks, J. Meyer, H. Bechtel, D. Wiechert, R. Mueller-Mach, G. Mueller, W. Schnick, *Seventh Int. Conf. Solid State Light.* **2007**, *6669*, P6690–P6690.

[50] K. Uheda, K. Uheda, S. Shimooka, S. Shimooka, M. Mikami, M. Mikami, H. Imura, H. Imura, N. Kijima, N. Kijima, *Sci. Technol.* **2007**, 899–902.

[51] H. A. Höppe, H. Trill, B. D. Mosel, H. Eckert, G. Kotzyba, R. Pöttgen, W. Schnick, *J. Phys. Chem. Solids* **2002**, *63*, 853–859.

[52] S. Azam, S. A. Khan, R. Khenata, G. Murtaza, S. Bin Omran, S. Muhammad, *Zeitschrift fur Naturforsch. - Sect. A J. Phys. Sci.* **2015**, *70*, 897–904.

[53] H. Huppertz, Strukturelle Erweiterung der Nitridosilicate, doctoral thesis, Universität Bayreuth, **1997**.

[54] J. Rodriguez-Carvajal, *Fullprof. 2k-Version 4.40*, **2008**.

[55] J. F. Bérar, P. Lelann, *J. Appl. Crystallogr.* **1991**, *24*, 1–5.

[56] H.-P. Müller, R. Hoppe, *Zeitschrift für Anorg. und Allg. Chemie* **1992**, *611*, 73–80.

[57] *The Term Dreier Ring Was Coined by Liebau and Is Derived from the German Word “Drei”, Which Means Three; However, a Dreier Ring Is Not a Three-Membered Ring, but a Six-Membered Ring Comprising Three Tetrahedral Centers (Si) and Three Electronegative Atoms*, **n.d.**

[58] C. Braun, L. Mereacre, T. Stürzer, B. Schwarz, *Phys. status solidi* **2024**, *261*, 2300480.

[59] P. Dorenbos, *J. Lumin.* **2003**, *104*, 239–260.

[60] O. M. ten Kate, Z. Zhang, P. Dorenbos, H. T. Hintzen, E. van der Kolk, *J. Solid State Chem.* **2013**, *197*, 209–217.

[61] P. Dorenbos, *J. Phys. Condens. Matter* **2005**, *17*, 8103–8111.

[62] P. Dorenbos, *J. Mater. Chem.* **2012**, *22*, 22344–22349.

[63] O. M. Ten Kate, Z. Zhang, J. R. Van Ommen, H. T. Hintzen, *J. Mater. Chem. C* **2018**, *6*, 5671–5683.

[64] G. Blasse, B. C. Grabmaier, **1994**, DOI 10.1007/978-3-642-79017-1.

[65] C. B. Palan, N. S. Bajaj, S. K. Omanwar, *Mater. Res. Bull.* **2016**, *76*, 216–221.

[66] S. H. M. Poort, H. M. Reijnhoudt, H. O. T. Van Der Kuip, G. Blasse, *J. Alloys Compd.* **1996**, *241*, 75–81.

[67] F. M. Ryan, W. Lehmann, D. W. Feldman, J. Murphy, *J. Electrochem. Soc.* **1974**, *121*, 1475.

[68] J. W. H. Van Krevel, H. T. Hintzen, R. Metselaar, A. Meijerink, *J. Alloys Compd.* **1998**, *268*, 272–277.

[69] X. Yang, X. J. Xing, Y. F. Liu, C. H. Mu, H. Van Bui, Z. W. Zhang, S. Agathopoulos, X. Xu, L. J. Yin, *Opt. Mater. (Amst).* **2021**, *121*, 111506.

[70] H. A. Höppe, H. Lutz, P. Morys, W. Schnick, A. Seilmeier, *J. Phys. Chem. Solids* **2000**, *61*, 2001–2006.

[71] Q. Wang, Y. Dong, Q. Shao, X. Teng, J. Jiang, H. T. (Bert. Hintzen, *Opt. Mater. (Amst).* **2017**, *66*, 314–318.

[72] W. Chuang, X. Shuangyu, W. Xicheng, Z. Ge, W. Quansheng, W. Yuhua, *New J. Chem.* **2015**, *39*, 6958–6964.

[73] X. Song, R. Fu, S. Agathopoulos, H. He, X. Zhao, X. Yu, *J. Am. Ceram. Soc.* **2011**, *94*, 501–507.

[74] H. Lutz, S. Joosten, J. Hoffmann, P. Lehmeier, A. Seilmeier, H. A. Höppe, W. Schnick, *J. Phys. Chem. Solids* **2004**, *65*, 1285–1290.

[75] M. Göppert‐Mayer, *Ann. Phys.* **1931**, *401*, 273–294.

[76] W. Kaiser, C. G. B. Garrett, *Phys. Rev. Lett.* **1961**, *7*, 229.

[77] W. Schnick, H. Huppertz, R. Lauterbach, *J. Mater. Chem.* **1999**, *9*, 289–296.
